# Supplementary figures and images for: Plasmacytoid dendritic cells have divergent effects on HIV infection of initial target cells and induce a pro-retention phenotype
Source: PLoS Pathog. 2021 Apr 19;17(4):e1009522. doi: 10.1371/journal.ppat.1009522 (PMC8084337; doi:10.1371/journal.ppat.1009522)

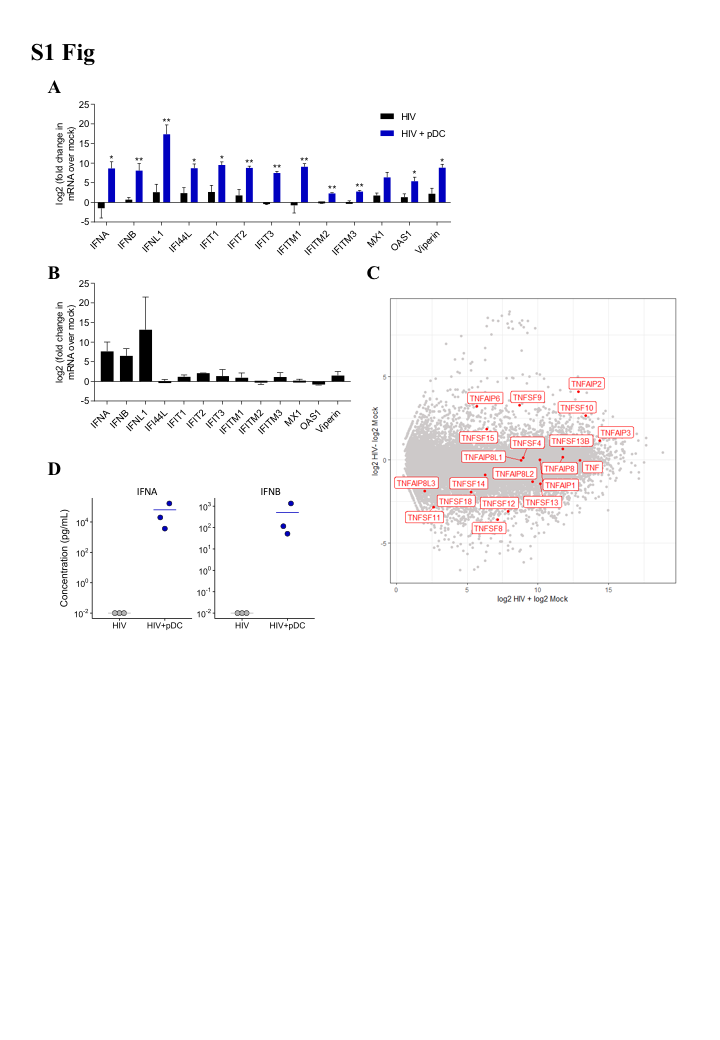

Supplement: S1 Fig — (S1A) pDCs induce IFN and ISG expression in MDDC-pDC cocultures. Gene bank accession numbers can be reviewed in Nasr et al (3). n = 4, *p < 0.05, **p < 0.01 by paired two-sided t-test with multiple testing correction. (S1B) HIV exposed pDCs expressed IFNs but no significant ISGs mRNA. (S1C) RNAseq data showing TNF subtype gene induction in pDCs at 18 hours post HIV exposure. (S1D) Detection of IFNα and IFNβ by ELISA in supernatants derived from MDDC-pDC cocultures. (TIF) [file ppat.1009522.s001.tif]

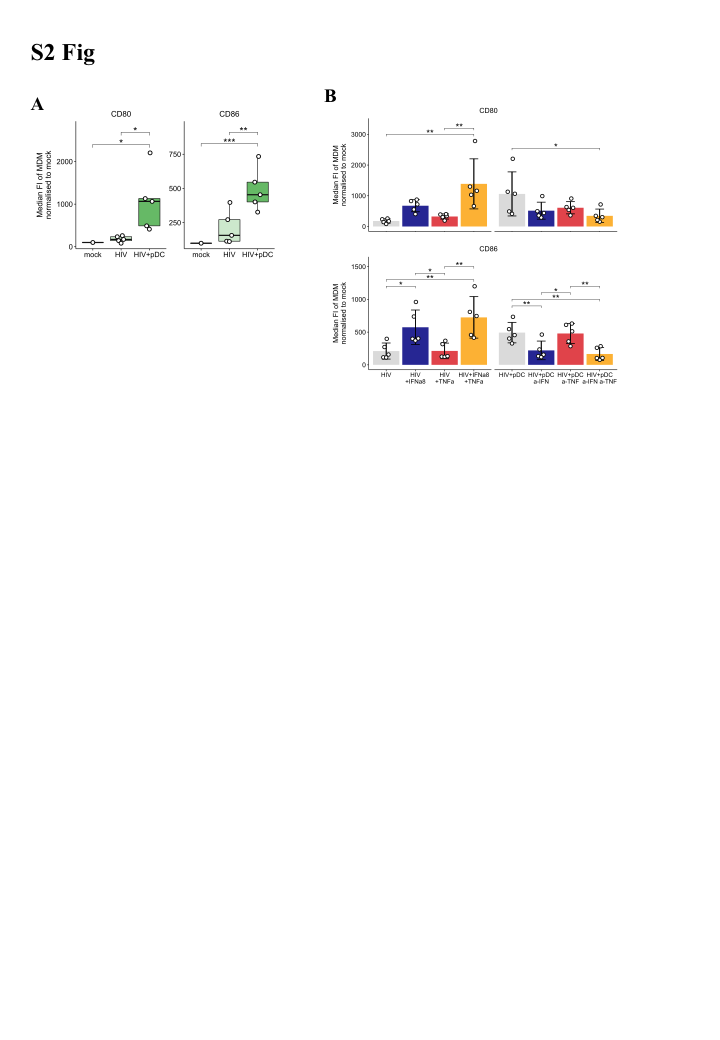

Supplement: S2 Fig — (S2A-B) Induction of maturation marker on MDMs cocultured with pDCs. The median FI of maturation markers CD80 (B-upper panel) and CD86 (B-lower panel) in MDDCs that were either mock, HIV infected MDDCs in the presence and absence of pDCs, HIV infected MDDCs treated with exogenous rIFNα8 and/or rTNFα, HIV infected MDDCs treated with antibodies to blocking IFN and/or TNF signaling in pDC cocultures. Data is shown as normalized to 100% in mock infected cells. n = 5 individuals, *p < 0.05, **p < 0.01, ***p < 0.001 by repeated measures ANOVA with Tukey post-hoc test. (TIF) [file ppat.1009522.s002.tif]

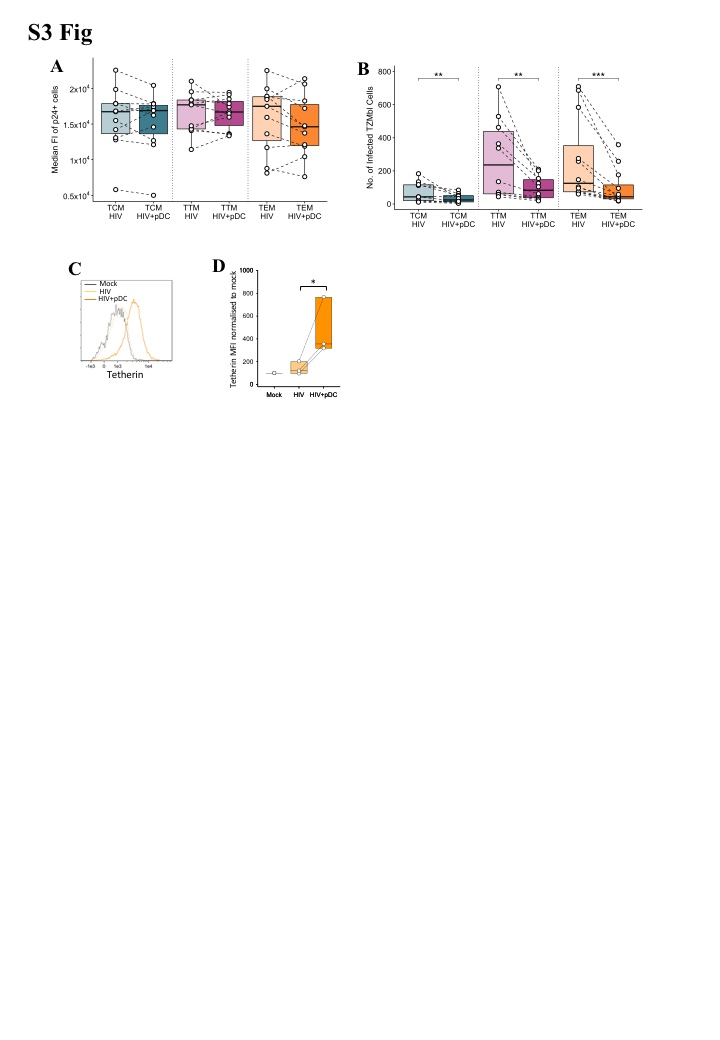

Supplement: S3 Fig — (S3A) Median fluorescent intensity (FI) of p24+ TCM, TTM and TEM (n = 10) in the absence (HIV) or presence of pDCs (HIV+pDC). (S3B) Assessment of infectious virus release via the TZMbl assay. Graphs show the number of infected cells after exposure to infectious supernatants derived from infected resting TCM, TTM and TEM cells in the absence (HIV) or presence of pDCs (HIV+pDC). n = 10–11 individuals, **p < 0.01, ***p < 0.001 by Wilcoxon signed rank test. (S3C-D) Quantification of Tetherin expression by Imagestream flow cytometry in TEM that were either mock or infected with HIV in the presence and absence of pDCs. n = 3 *p < 0.05 by one tail paired T test. (TIF) [file ppat.1009522.s003.tif]

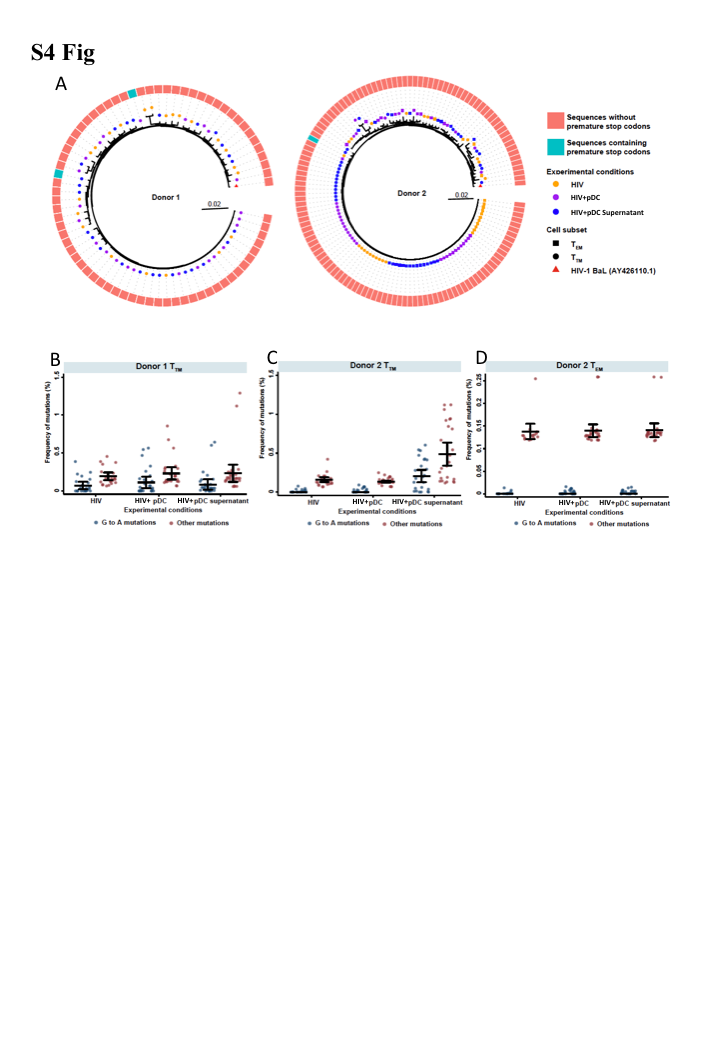

Supplement: S4 Fig — (S4A) Phylogenetic trees of HIV-DNA sequences within CD4 memory T-cells derived from Donor 1 and Donor 2. For the phylogenetic trees, individual HIV-DNA sequences derived from the memory T-cells infected with HIV-1 BaL (HIV) and the sequences from the infected cells cocultured with pDC (pDC) or supernatant from pDC (Supernatant from pDC) are shown as in the legend. For Donors 1 and 2, HIV-DNA sequences derived from TTM cells were included in the tree (circle). For Donor 2, the viral sequences isolated from TEM cells are shown (square). The outer layer of each phylogenetic tree shows the sequences containing premature stop codons (green square). The sequences without the stop codons are also indicated in this outer layer (pink square). HIV-1 BaL (accession: AY426110.1) was used to root the trees (red triangle). (S4B to D) Frequencies of G to A mutations (blue data points) and non-G to A mutations (pink data points) within individual HIV-DNA sequences obtained from the infected TTM cells (Donors 1 and 2) and TEM cells (Donor 2) exposed to the three experimental conditions. (TIF) [file ppat.1009522.s004.tif]

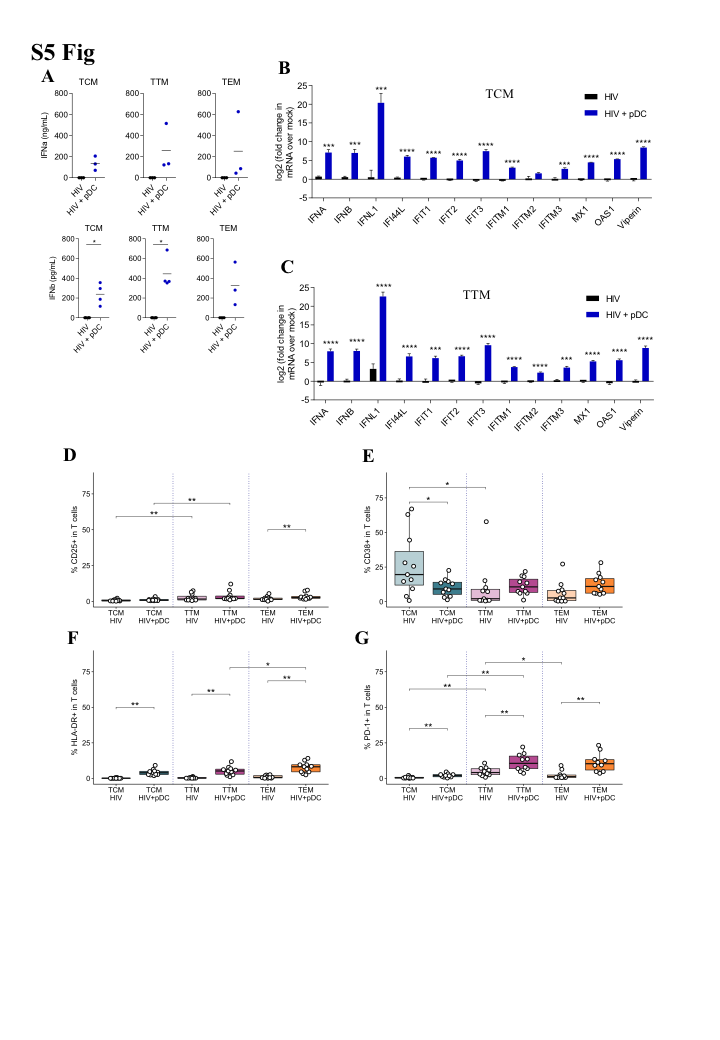

Supplement: S5 Fig — (S5A) Detection of IFNα and IFNβ by ELISA in supernatants derived from T cells -pDC cocultures. (S5B-C) pDCs induce IFN and ISG expression in TCM (B) and TTM (C) cocultured with pDC. n = 3 *p < 0.05, **p < 0.01 by paired two-sided t-test with multiple testing correction. (S5D-E) Expression of T cell activation markers. Graphs show the percentage of resting T cells expressing CD25 (S5D), CD38 (S5E), HLADR (S5F) and PD1 (S5G) in the absence (HIV) or presence of pDCs (HIV+pDC). n = 11 individuals, *p < 0.05, **p < 0.01, ***p <0.001 by one-way repeated ANOVA. (TIF) [file ppat.1009522.s005.tif]

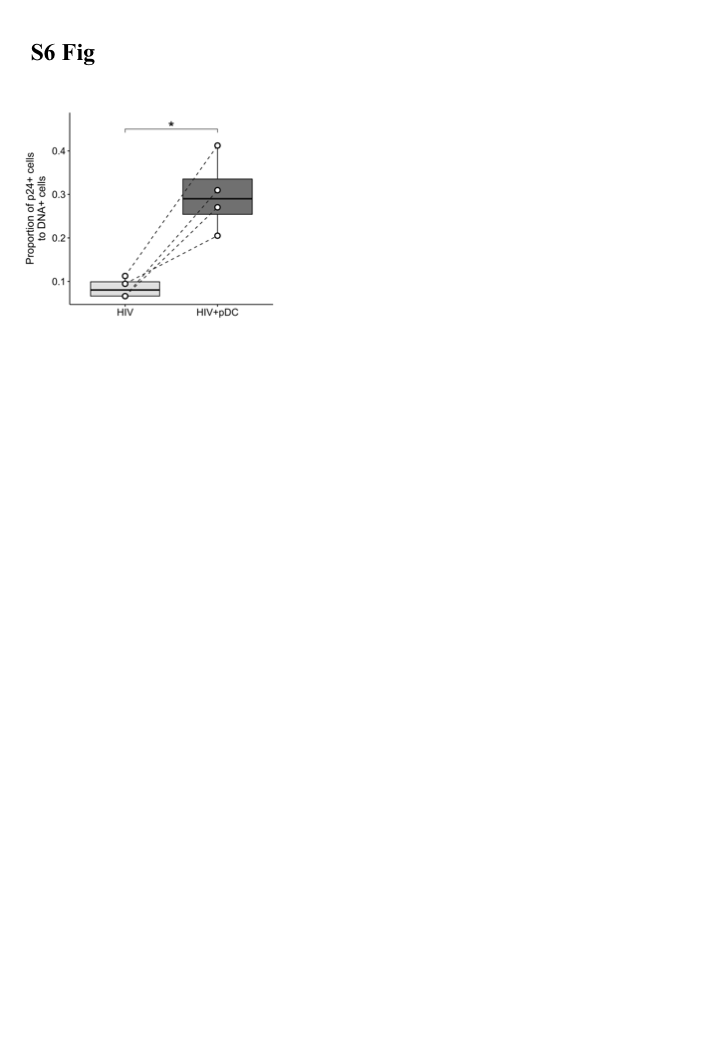

Supplement: S6 Fig — *p < 0.05 by paired two-sided t-test. (TIF) [file ppat.1009522.s006.tif]

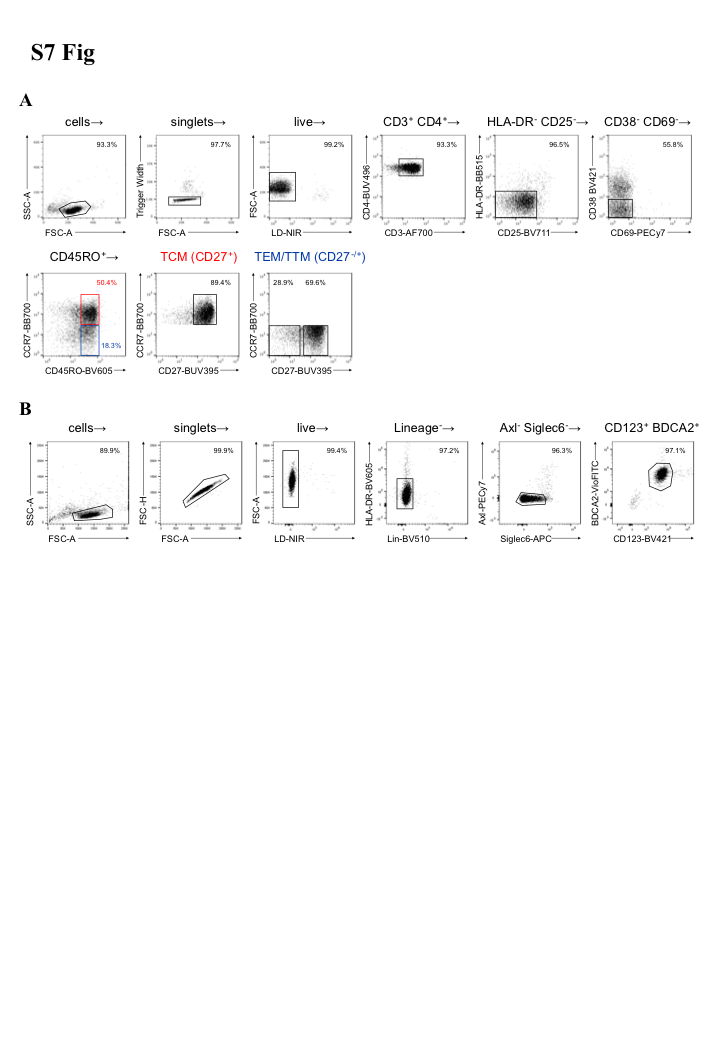

Supplement: S7 Fig — (S7A) Cell sorting strategy to isolate resting memory CD4 TCM, TTM and TEM cells from blood. After excluding debris, doublets and dead cells, live CD3 CD4 T cells were gated. Resting cells were identified as the negative population of cells that did not express the T cell activation markers HLADR, CD25, CD38 or CD69. Memory cells were gated as CD45RO+ with CCR7 and CD27 then used to discriminate between the TCM (CCR7+ CD27+), TTM (CCR7- CD27+) and the TEM (CCR7- CD27-) subsets. (S7B) Cell Sorting Strategy to isolate ‘bona-fide’ pDCs from blood. By excluding Axl+ Siglec6+ cells, ‘bona-fide’ pDCs in our co-cultures were Lineage−HLA-DR-/lo Axl− Siglec6– BDCA2+ CD123+ cells with purity >95%. (TIF) [file ppat.1009522.s007.tif]
