# Supplementary material for: Plasmacytoid dendritic cells have divergent effects on HIV infection of initial target cells and induce a pro-retention phenotype
Source: PLoS Pathog. 2021 Apr 19;17(4):e1009522. doi: 10.1371/journal.ppat.1009522 (PMC8084337; doi:10.1371/journal.ppat.1009522)
Supplement: Attachment — Submitted filename: Response to reviewers.docx [file ppat.1009522.s008.docx]

Thank you for your insightful comments, we appreciate all the time and effort the editorial

members and reviewers have contributed to the improvement of this manuscript.

Below are our responses to each comment in grey font and what have been included in the manuscript is in red font.

**Part II – Major Issues: Key Experiments Required for Acceptance**

**Reviewer #1**

***1)*** *One of the main messages of the resting CD4+ T cell competent of the study is that pDC (through their production of IFN) reactivate latent infection (evident by an increase in % of p24+ cells even after blocking viral entry). While the results presented in the study may support this hypothesis (especially in light of recent literature), a couple of potential experiments could strengthen this point, including coculture pDCs with latently infected cells (from latency models such as JLat or ACH2 or CD4+ T cells from people living with HIV on suppressive ART) to directly measure latency reactivation mediated by pDCs. Another possibility would* *be sort p24-negative cells and only coculture these with pDCs and observe latency reactivation.*

**Response:** As the Reviewer requests we have now shown reversal of viral latency using J-Lat cells (clone 10.6). This is now added to page 16 lines 336-340 and as Fig 5J-K to read:

Finally, to confirm that HIV reactivation from latency was mediated by pDCs, we used the latently infected J-Lat cells (clone 10.6) and exposed them to supernatants derived from infected TEM-pDC co-cultures or co-cultured them with pDCs. At 2 dpi, we detected a significant increase in HIV GFP expression indicating that pDCs are indeed reactivating latency in J-Lat (Fig 5J-K). We have also amended the Fig 5 legend to include J-K.

*Finally, the MFI of all of these experiments should be presented in addition to % of p24. The MFI should probably also increase per cell basis if pDCs have a reactivation ability.*

**Response:** We think MFI is not as important as % p24 in determining reactivation for the following reason: % p24 measures a qualitative change from no p24 expression to detectable p24. The p24- cells are either not infected or latently infected, whilst the p24+ cells are undergoing partial or full HIV replication. Unless pDCs block the post-transcriptional/translational process following reactivation, there should be no change in MFI. Nevertheless, we have examined this and included “there was no change in the number of virions per cell” on page 10 line 199 and showed this as supp S3A.

***2)*** *Another important observation was the reduction in viral infectivity (in supplemental figures), matching with an APOBEC activity in mutating progeny virus and rendering them defective This observation could be added to the main Figures, and the authors could sequence this virus to show if it has an APOBEC G-A mutation signatures. That can add depth to this important point that can highlight the opposing effects of IFN and pDC (on the one hand, reactivate the virus but render some of them defective). Also, the increase in viral expression that does not translate to an increase in viral production is consistent with BST2 (tetherin) activity. Again, quick staining of (tetherin on the same cells, if available, could add more depth to this observation.*

**Response:** The reviewer has mentioned two important ISGs, APOBEC3G and Tetherin, induced by IFN that can inhibit HIV replication and spread and might be responsible for reduction in extracellular viral production. As requested, we have now examined APOBEC and Tetherin expression, their effects and determined that there were two mechanisms of reduced viral spread despite enhance reactivation.

**1. Reduction in extracellular viral infectivity** due to IFN presence in the coculture supernatants used to infect TZMBL, as expected. This simulates the pre-treatment of cells with IFNα for 24 hours prior to HIV exposure which has been shown to potently reduce HIV replication (1), although our TZMBL were exposed simultaneously to HIV and IFN upon their exposure to the viral supernatants.

**2. Restriction of viral egress by Tetherin:** When we measured both extracellular live and defective virion concentrations by both p24 ELISA and reverse transcriptase (RT) we showed that there was a no increase in extracellular virus production in the presence of pDCs despite the marked and significant increase in intracellular p24 expressing CD4 T cells. We investigated whether this is due to Tetherin and we used two approaches to examine Tetherin expression in TEM in three experimental conditions: mock, HIV and HIV+ pDC via i) Imagestream flow cytometry for quantification and ii) deconvolution fluorescent microscopy (Deltavision) for quantifying individual Tetherin punctae, their intensity of staining within the cells and colocaliasation with p24. We have now added to the results on p11 lines 214-223. ‘This is consistent with two effects, an effect of IFN inducing late acting ISGs such as Tetherin. Upon investigation by Imagestream flow cytometry, we found a baseline level of Tetherin expression in mock infected memory T cells which was similar in HIV infected cells but was markedly and significantly increased increased upon the addition of pDCs to these infected cells (S3C-D Fig). Immunoflorescent microscopy showed wide distribution of tetherin in the CD4 T cell cytoplasm with sub-cell membranous foci with all treatments. However, with addition of pDCs to HIV infected cells, there was a marked and significant increase in colocalization between p24 and Tetherin of 24% compared to only 8% in HIV infected cells in the absence of pDCs. This colocalization was mostly at the cell surface, and thus likely to reduce the release of budded HIV-1 virions from infected cells (Fig 3H-I)’.

We have also added to the discussion on page 22 line 491-495: ‘This is consistent with the increased Tetherin expression and submembranous colocalization with p24 in memory CD4 T cells induced by IFNα secreted by pDCs (Fig 3H-I). This increased expression and colocalization of Tetherin would reduce the release of budded HIV-1 virions from infected cells (55, 56) and therefore inhibit further CD4 T cell infection and viral spread.’

Thus Tetherin induction is likely to have significantly impeded the release of the HIV virions and will be further explored by knockdown experiments in the future to determine its relative importance compared to other undefined mechanisms.

**APOBEC3G:** Thank you for this suggestion, we investigated whether APOBEC3G mediated A to G hypermutation played a role in reducing viral infectivity. Prof Sarah Palmer and her group performed single-proviral sequencing to obtain individual HIV-DNA *env* V1-V3 sequences from CD4+ memory T-cells infected with HIV-1 BaL and cocultured with either pDC or the supernatant of pDC. We found no evidence for G to A hypermutation within the viral sequences isolated from the TTM and TEM cells which were infected with HIV and then cocultured with either pDC or the supernatant of pDC. The viral sequences containing premature stop codons were rare within the memory T-cells alone or those cocultured with pDC or the supernatant of pDC. In addition, the frequency of G to A mutations within the viral sequences was significantly lower than the non-G to A mutations across all experimental conditions. Therefore, our sequencing analysis shows little evidence for the APOBEC3G mediated hypermutation elicited by pDC. We added to results p11-12 line 224-241 ‘Another intracellular antiviral mechanism is APOBEC3G mediated G to A hypermutation which causes stop codons in HIV-DNA during reverse transcription preventing further HIV replication and infection (28, 29). To determine if the presence of pDC enhances APOBEC3G hypermutation of HIV, we performed single-proviral sequencing to obtain individual HIV-DNA *env* V1-V3 sequences from TTM and TEM infected with HIV-1 BaL and cocultured with either pDC or the supernatant of pDC. We found no evidence for G to A hypermutation within the viral sequences isolated from the TTM and TEM cells which were infected with HIV and then cocultured with either pDC or the supernatant of pDC. The viral sequences containing premature stop codons were rare within the memory T-cells alone or those cocultured with pDC or the supernatant of pDC (S4 Fig). In addition, the frequency of G to A mutations within the viral sequences was significantly lower than the non-G to A mutations across all experimental conditions (S4B-D Fig.; p-value: <0.0001-0.037). Therefore, our sequencing analysis shows little evidence for the APOBEC3G mediated hypermutation elicited by pDCs, suggesting little effect in exiting virus. In summary there was restriction of viral egress from HIV infected cells when cocultured with pDCs, despite enhanced cytoplasmic p24 expression, probably partly by tetherin. The second effect was a further decrease in extracellular virus infectivity and spread probably soluble factors, especially type I IFN, secreted by pDCs’.

We have also added to the discussion on page 23 line 495-499 ‘The reduction in extracellular virus infectivity was unlikely to be due to IFNα induced APOBEC3G as our sequencing analysis showed no evidence for APOBEC3G mediated hypermutation in proviral HIV DNA elicited by pDCs. Rather the reduction in infectivity was likely due to the direct antiviral effect of type I interferons on the adjacent cells’.

***3)*** *While the manuscript focused on highlighting early infection events, an important potential implication of it could be during therapy interruption. Recently, it has been shown (Julie L. Mitchell et al., JCI (Plasmacytoid dendritic cells sense HIV replication before detectable viremia following treatment interruption)) that pDC production of IFN inversely correlates with time to viral rebound after treatment interruption. I think that deserves more discussion and how the current data align (or not) with this observation.*

**Response:** The reviewer raises an excellent point, which is now included in the discussion on page 23-24 lines 511-518: …with a recent study (ref) monitoring the pDC response to initial low-level viral replication after ART interruption in HIV infected patients and thus mimicking the events of early acute infection, which is very challenging to measure. The IFN production by pDCs was inversely correlated with viral load and consistent with our data for both myeloid and lymphoid cells in vitro and strongly suggest that early IFN production by pDCs reduces HIV infection, and viral reservoir formation. Furthermore, LTNP with chronic low-level infection have higher numbers of pDCs compared to healthy noninfected controls (ref).

***4)*** *All infections were performed using HIV-Bal (CCR5 tropic) or a transmitted-founder virus that is also CCR5 tropic. It would have been good to repeat some key experiments with a CXCR4-tropic virus (such as NL43) and show whether the results (for both myeloid or CD4+ T cells are tropic-dependent or not.*

**Response:** We did not investigate R4 infection of DCs and macrophages as we are modelling pDC influence on HIV infected cells in mucosa during sexual transmission where i) only R5 strains are more frequently transmitted (*2*); ii) the mucus in the female genital tract provides a protective barrier to CXCR4 infection (*3*); iii) immature myeloid DCs are preferentially infected by R5 virus (*4-7*) while mature DCs are not susceptible to infection by either X4 or R5 but they can transmit X4 to T cells via first phase (*8*). Based on this, we appropriately used BaL and a primary transmitted founder virus in myeloid cells and CD4 T cells. X4 strains emerge later in infection and may be more relevant in lymph nodes and secondary lymphoid tissues. We have started to look at X4 viruses’ infection in CD4 T cells and a future paper will focus on this aspect.

**Reviewer #2**

*The pDC used for the cocultures were previously frozen. While this was done to allow culture with autologous cells, it is known that PDC decrease in function and viability during cryopreservation. It is not clear if fresh PDC would not have an even stronger or distinct effect. The viability and capacity of IFN-I production of fresh and frozen PDC as used in the protocol could be compared or other control experiments could be performed with fresh PDC, such as testing the impact of supernatants from fresh PDC on infected cells. And could it be that some of the effects are mediated by factors released from dying PDC?*

**Response:** We do acknowledge that frozen pDCs may have had a slightly reduced function compared to fresh ones, but we mitigated that by freezing pDCs in CryoStor® 10 (CS10) rather than normal media supplemented with 10% DMSO. CS10 provides enhanced cell viability and functionality. After pDCs were thawed, they were rested for 1-2 hours at 37 ^0^C before cell sorting, with a pre-sort viability of 90-95% and exclusion of any dead cells following cell-sorting. IL-3 was added to pDC-T cell cocultures to promote their viability (now mentioned in Methods page 29 line 643-644), with median pDC viability on day 3 post co-culture or HIV infection being 88% (IQR 70-90%) as assessed by flow cytometry viability stain. Therefore, the observed effects were **not** mediated by factors released from dying PDCs. In addition, when were used fresh allogeneic pDCs in T-pDC cocultures (n=6), we detected a similar magnitude of increased p24 in T cells as in frozen autologous co-cultures with no statistical difference. However, CD69 induction was induced at a much higher level in allogenic cocultures due to the mixed lymphocyte reaction (MLR) nature of the experiments (added to results on page 15 lines 312-316). Therefore, freezing autologous pDCs does not affect our results . We have also assessed different ratios of autologous frozen pDCs to T cells ranging from 1:1; 1:2, 1:3, 1:4, 1:5, 1:10, 1:20 and found that all ratios up to 1:10 upregulated p24 and CD69 to similar levels indicating that there may be a minimum amount of IFNα production required to induce the observed effects. In the future, we will treat infected T cells with different concentrations of IFNα to assess the minimal concentration required to reach saturation of HIV reactivation and CD69 induction.

*The study is also interesting because it tries to reproduce the physiological conditions by using the IFN-I-producing cells instead of adding exogeneous IFN-I to the cultures. Still, very high MOI were used to infect the cells (probably in order to reach a high frequency of infected cells and to be able to detect intra-cellular p24). Such high MOI might in most cases not correspond to physiological conditions and might hide a potential inhibitory effect of the PDC in the CD4 T cells. It would be good to confirm some data with lower MOI. The reduction of viral replication might be measured in that case by more sensitive methods (RT-PCR or p24 Simoa).*

**Response:** **i)** Resting CD4+ T cells are known to be relatively resistant to HIV-1 infection compared to activated T cells and therefore delivering a higher number of viral particles increases the proportion of resting cells that become infected. In support of this, other studies have used MOI of 3 (*9*) to infect resting CD4 T cells and MOI of 1 (*10*) to infect activated primary CD4+ T cells. **ii)** As shown in this paper, within the different subsets of resting T cells, HIV infection or DNA integration is much lower in TCM compared to TTM and TEM. Therefore, we have chosen the MOI of 0.75 based on the viral titration of different MOIs (0.5, 0.75 and 1) in PHA/IL-2 activated CD4 T cells and the resting TCM cell subset. At 72 hours pi, when we compared MOIs of 0.5 and 0.75, we observed increased p24 expression in the activated CD4+ T cells and TCM cells. However, p24 expression plateaued with the MOI of 0.75 and 1 in both the activated CD4+ T cells and the resting TCM cells. When MOIs below 0.75 were used, we did not detect any p24 infection in TCM derived from 75% of our donors. As HIV infection and DNA integration is much lower in TCM compared to TTM and TEM, the MOI of 0.75 was used for the subsequent infection of T cells (this has been added to materials and methods page 28-29 lines 626-634) which is far below the MOI of 3 used in other studies. **iii)** Despite the very low levels of HIV infection in some donors we still detected the same patterns of increased intracellular p24 across all resting T cell subsets. This implies that the MOI used in our study did not hide any potential inhibitory effect of the pDC on the CD4 T cells.

**Part III – Minor Issues**

**Reviewer #1:**

***5)*** *A minor point, it is not clear how the statistical analysis in Figure 4C was done; if it was a simple correlation, that would have been wrong as repeated measures of same sample donors were used in the plot (different subpopulations of same donors), and that should be adjusted in the statistical analysis. The correlation will obviously stay highly significant, but the p-value might change for scientific accuracy.*

**Response:** We have now used a linear mixed model using the lme4 and lmer Test functions in R, treating subset as a fixed variable and added this to the stat section on page 35 lines 797-799

**6)** *Another minor point, having the methods following the results, the S1A Fig. should be moved to be the last supplementary Figure as it first appears in the methods after all other supplementary Figures.*

**Response:** We have now amended S1 to be the last supplementary figure S7 and subsequently change the chronological order of all supplementary figures as they appear in the text.

**Reviewer #2**

*The lack of detectable productive infection of pDC might be related to low viral production or to the fact that pDC were cultured apparently for 3-5 days, which might not be sufficiently long for such cells to identify productive infection, in particular if measured by intra-cellular p24 that is not a very sensitive method. Also, pDC do not survive a long time in culture. In addition, it seems that the PDC were frozen, which likely has resulted in reduced viability of the cells. The statement that there was no productive infection in pDC should be moderated and complemented by these limitations for clarification.*

**i) No productive infection in pDC:** Fresh pDCs and pre-DCs (also known as AXL^+^Siglec^+^ myeloid DCs (ASDCs)) were recently separated phenotypically and transcriptionally (*11, 12*) and then exposed to an MOI of 0.1 of an R5 utilising strain (*13*). At 48 hours post HIV exposure, pre-DCs were shown to be infected (4.2 ± 2.5%) due to their Siglec-1 expression that mediates efficient HIV-1 capture and infection while pDCs remained refractory to infection (Fig. 1 C and D in Ruffin et al., 2019). In addition, this paper also showed that pre-DCs transfer HIV to T cells while pDCs did not. Therefore, on page 8 lines 137-140 we amended no productive infection to “No p24+ pDCs” and added This was similar to a recent study showing that when authentic pDCs are separated from ASDCs they cannot be infected with HIV and cannot transfer virus to CD4 T cells (*13*). Previous studies showing pDC infection did not exclude pre-DCs (*14*) that express CD123 like pDCs.

**ii) Survival of pDCs after freezing:** Please refer to response on page 4.

*Lines 212-220: It was analysed if the increased p24 expression in memory CD4+ T cells cocultured with pDC was not due to a defect in IFN production and/or ISG expression. The results are presented as “data not shown”. The results could be shown as supplementary data as this seems to be an important point.*

**Response:** We have now included this as supplementary Fig S5A-C.

*Can it be excluded that the decrease of integrated HIV DNA is not the result of proliferation (and in consequence of a dilution of infected cells within total cells) and/or of more de novo infections (which are generally characterized by a higher ratio of non-integrated versus integrated DNA)?*

**Response:** We extended the co-culture conditions from 5 to 10 days post infection to assess whether pDCs modulate CD4 T cell proliferation using both Cell Trace FarRed and an anti-Ki76 that targets the proliferation marker protein Ki-67. We did not observe cell proliferation with both approaches in the presence of pDCs. This is consistent with the recently attributed functions of authentic pDCs (used in our study) as having a weak T cell stimulatory potential while ASDCs or pre-DCs are potent inducers of T cell proliferation (*11, 12*).

We have also measured the copy number of integrated HIV DNA versus non-integrated DNA (2-LTR circles) in HIV-infected TCM, TTM, TEM and activated T cells at 72 hours post infection which correspond to approximately 3 rounds of infection. As shown in Fig 1 (page 8 of this response letter), the number of 2-LTR circles in each cell type was lower than the integrated copies indicating that there significantly more integrated than non-integrated DNA. Our observation is similar to another study showing that non-integrated copies are always lower in clade A, B, E, AE but not in C following in vitro infection of pre-stimulated PBMCs (*15*). Therefore, based on the above, we added on page 20 lines 425-427 “The decrease in integrated HIV DNA was not due to cell proliferation as we did not detect any cell proliferation using both Cell Trace FarRed and the proliferation marker protein Ki-67”.

*Can the authors show examples of the DNA scope images?*

**Response:** Previous Fig 5H is now Fig 5I and we included the DNAScope images as Fig 5H.

*IFN-α/β is already known as a strong inducer of the cell surface activation marker, CD69, and thereby promoting lymphocyte retention in lymphoid organs via S1P1 (Shiow et al, Nature 2006). The fact that CD69 can be considered as an ISG might we worth to be added in the discussion. (Shiow et al, Nature 2006; doi: 10.1038/nature04606; Renson et al Vet Res 2010).*

**Response:** We added to the discussion on page 20 line 434 Shiow et al as a ref and on page 21 lines 447-449 “ As CD69 was previously shown to be directly induced by IFNα/β (Shiow, 2006; Renson, 2010), it may be also considered as an ISG, in addition to its activation and tissue retention characteristics.

*There is little discussion on the mechanisms of how IFN-I could mediate higher intracellular p24. It has indeed been shown that paradoxically, viral production can be enhanced by IFN-I (Seo et al, Science 2011). IFN-I regulates glucose metabolism (Burke et al, JVI 2014, doi: 10.1128/JVI.02649-13). HIV is expressed preferentially in CD4+ T cells with high glycolytic activity that are also characterized by high levels of ISGs (Valle Casuso et al, Cell Metabolism, DOI:*[*https://doi.org/10.1016/j.cmet.2018.11.015*](https://doi.org/10.1016/j.cmet.2018.11.015)*). HIV-1, as many other viruses, thus exploits the metabolic environment for its replication cycle, and this might be a way to evade some restriction factors. Could it be that the pDC impacted the metabolic state of the CD4+ T cells through IFN-a? Would it be possible to analyse markers for higher metabolic/glycolytic activity in the co-cultured CD4+ T cells, such as Glut1 expression? (Loisel-Meyer et al, PNAS 2012).*

**Response:** As suggested by the reviewer we had commenced investigating the mechanism of HIV reactivation by pDCs and type I IFN using single cell RNA sequencing and comparing our data to other studies using Latency Reactivating Agents. So far, we have found some interesting and relevant differential gene expression, likely coding for proteins which interact with Tat and vpu, interface with NF-κb, enhance transcriptional elongation and code for long non-coding RNAs which may modulate HIV infection. Approximately 20 of our induced genes overlap with the genes induced by different LRAs (Coelho et al, 2020) while others (~50) do not (NB We have used several methods of analysis particularly to evade the hundreds of genes induced by interferon which are irrelevant to HIV reactivation and increased p24 expression). This is the basis of a large continuing and separate study beyond the scope of the current paper, as it requires extensive downstream analysis to differentiate genes/proteins involved in initial reactivation from those facilitating replication and to determine the relative importance of these induced genes. However, we have mentioned this approach and preliminary findings on page 24 line 529-540 of the discussion.

Although the reviewer makes some excellent suggestions about potential mechanisms of pDC/IFN induction of HIV reactivation in memory T cells, especially the effects on metabolic activity and glucose uptake for both HIV de novo and latent infection, as reported by Valle-Casuso et al., Glut1 expression was not prominent amongst our differentially expressed gene set. Nevertheless we added to the discussion on page 19 lines 406-4111: Valle Cusso et al, showed that glucose transporter-1 (Glut1) expression increases from naïve to TCM and TEM and that the susceptibility of CD4 T cell subsets to HIV infection matches their Glut1 expression and metabolic activity as HIV selectively infects highly metabolic CD4+ T cells, independent of their activation phenotype (Valle Cusso et al). This correlates with the differential and increased level of HIV infection we observed in CM, TM and EM in the absence of pDCs. We also added on page 24 line 538-540 of the discussion “Although our transcriptomic studies did not show any Glut RNA signal, this potential mechanism for reactivation will be examined in future”.

*From Line 403-405: while the increase of HLA-DR was less than for CD69, the HLA-DR increases on the CD4+ T cells after co-culture with PDC were still significant and consistently seen (Fig.S5C). It is thus not convincing to conclude that “the cells are maintained in a predominantly resting state” as some activation probably still have occurred. This does not change the overall conclusions of the study.*

**Response:** We agree that there were significant increases in HLA-DR and CD25 expression on HIV infected T cells in the presence of pDCs (and we state this in the text on page 13 line 258-259) but this did not exceed 10%. ie 90% of cells were in resting state. This is consistent with our statement that ‘cells are maintained a predominantly resting state’. We agree this does not change the conclusions of the study as the proportion of cells expressing CD69 far exceeded those expressing CD25 and HLA-DR

We hope the paper is now acceptable for publication.

Yours sincerely,

Dr. Najla Nasr and Professor Tony Cunningham

On behalf of all Authors

Figure 1. Detection of HIV integration and 2-LTR circles. TCM, TEM, TTM and activated CD4+T cells were infected for 72 hours with the HIV, and nested PCRs were performed to determine the copy number of integrated HIV DNA, 2-LTR circles and CD3 gene. HIV copy numbers per 1x 10^6^ cells were determined by dividing the copy number of either HIV integration or 2-LTR by the CD3 copy number, and multiplying this by 2 x 10^6^, as there are two copies of the CD3 gene per cell. For integration TCM n=6; TTM n=5; TEM n=3 while for 2-LTR TCM n=3; TTM n=3; TEM n=3. For the activated CD4+ T cells, n=6 for integrated and n=3 for 2-LTR. Error bars represent the SEM. *p<0.05, **p<0.01 (Two-tailed Mann-Whitney test).

1.	D. Vendrame, M. Sourisseau, V. Perrin, O. Schwartz, F. Mammano, Partial inhibition of human immunodeficiency virus replication by type I interferons: impact of cell-to-cell viral transfer. *Journal of virology* **83**, 10527-10537 (2009).

2.	B. F. Keele, E. E. Giorgi, J. F. Salazar-Gonzalez, J. M. Decker, K. T. Pham, M. G. Salazar, C. Sun, T. Grayson, S. Wang, H. Li, X. Wei, C. Jiang, J. L. Kirchherr, F. Gao, J. A. Anderson, L. H. Ping, R. Swanstrom, G. D. Tomaras, W. A. Blattner, P. A. Goepfert, J. M. Kilby, M. S. Saag, E. L. Delwart, M. P. Busch, M. S. Cohen, D. C. Montefiori, B. F. Haynes, B. Gaschen, G. S. Athreya, H. Y. Lee, N. Wood, C. Seoighe, A. S. Perelson, T. Bhattacharya, B. T. Korber, B. H. Hahn, G. M. Shaw, Identification and characterization of transmitted and early founder virus envelopes in primary HIV-1 infection. *Proceedings of the National Academy of Sciences of the United States of America* **105**, 7552-7557 (2008).

3.	W. W. Agace, A. Amara, A. I. Roberts, J. L. Pablos, S. Thelen, M. Uguccioni, X. Y. Li, J. Marsal, F. Arenzana-Seisdedos, T. Delaunay, E. C. Ebert, B. Moser, C. M. Parker, Constitutive expression of stromal derived factor-1 by mucosal epithelia and its role in HIV transmission and propagation. *Curr Biol* **10**, 325-328 (2000).

4.	A. Blauvelt, H. Asada, M. W. Saville, V. Klaus-Kovtun, D. J. Altman, R. Yarchoan, S. I. Katz, Productive infection of dendritic cells by HIV-1 and their ability to capture virus are mediated through separate pathways. *The Journal of clinical investigation* **100**, 2043-2053 (1997).

5.	A. Granelli-Piperno, E. Delgado, V. Finkel, W. Paxton, R. M. Steinman, Immature dendritic cells selectively replicate macrophagetropic (M-tropic) human immunodeficiency virus type 1, while mature cells efficiently transmit both M- and T-tropic virus to T cells. *Journal of virology* **72**, 2733-2737 (1998).

6.	M. Pope, S. Gezelter, N. Gallo, L. Hoffman, R. M. Steinman, Low levels of HIV-1 infection in cutaneous dendritic cells promote extensive viral replication upon binding to memory CD4+ T cells. *The Journal of experimental medicine* **182**, 2045-2056 (1995).

7.	D. Weissman, Y. Li, J. Ananworanich, L. J. Zhou, J. Adelsberger, T. F. Tedder, M. Baseler, A. S. Fauci, Three populations of cells with dendritic morphology exist in peripheral blood, only one of which is infectable with human immunodeficiency virus type 1. *Proceedings of the National Academy of Sciences of the United States of America* **92**, 826-830 (1995).

8.	L. Ganesh, K. Leung, K. Lore, R. Levin, A. Panet, O. Schwartz, R. A. Koup, G. J. Nabel, Infection of specific dendritic cells by CCR5-tropic human immunodeficiency virus type 1 promotes cell-mediated transmission of virus resistant to broadly neutralizing antibodies. *Journal of virology* **78**, 11980-11987 (2004).

9.	M. J. Pace, E. H. Graf, L. M. Agosto, A. M. Mexas, F. Male, T. Brady, F. D. Bushman, U. O'Doherty, Directly infected resting CD4+T cells can produce HIV Gag without spreading infection in a model of HIV latency. *PLoS pathogens* **8**, e1002818 (2012).

10.	D. Zhou, T. Hayashi, M. Jean, W. Kong, G. Fiches, A. Biswas, S. Liu, H. O. Yosief, X. Zhang, J. Bradner, J. Qi, W. Zhang, N. Santoso, J. Zhu, Inhibition of Polo-like kinase 1 (PLK1) facilitates the elimination of HIV-1 viral reservoirs in CD4(+) T cells ex vivo. *Sci Adv* **6**, eaba1941 (2020).

11.	A. C. Villani, R. Satija, G. Reynolds, S. Sarkizova, K. Shekhar, J. Fletcher, M. Griesbeck, A. Butler, S. Zheng, S. Lazo, L. Jardine, D. Dixon, E. Stephenson, E. Nilsson, I. Grundberg, D. McDonald, A. Filby, W. Li, P. L. De Jager, O. Rozenblatt-Rosen, A. A. Lane, M. Haniffa, A. Regev, N. Hacohen, Single-cell RNA-seq reveals new types of human blood dendritic cells, monocytes, and progenitors. *Science* **356**, (2017).

12.	P. See, C. A. Dutertre, J. Chen, P. Gunther, N. McGovern, S. E. Irac, M. Gunawan, M. Beyer, K. Handler, K. Duan, H. R. B. Sumatoh, N. Ruffin, M. Jouve, E. Gea-Mallorqui, R. C. M. Hennekam, T. Lim, C. C. Yip, M. Wen, B. Malleret, I. Low, N. B. Shadan, C. F. S. Fen, A. Tay, J. Lum, F. Zolezzi, A. Larbi, M. Poidinger, J. K. Y. Chan, Q. Chen, L. Renia, M. Haniffa, P. Benaroch, A. Schlitzer, J. L. Schultze, E. W. Newell, F. Ginhoux, Mapping the human DC lineage through the integration of high-dimensional techniques. *Science* **356**, (2017).

13.	N. Ruffin, E. Gea-Mallorqui, F. Brouiller, M. Jouve, A. Silvin, P. See, C. A. Dutertre, F. Ginhoux, P. Benaroch, Constitutive Siglec-1 expression confers susceptibility to HIV-1 infection of human dendritic cell precursors. *Proceedings of the National Academy of Sciences of the United States of America* **116**, 21685-21693 (2019).

14.	A. Lederle, B. Su, V. Holl, J. Penichon, S. Schmidt, T. Decoville, G. Laumond, C. Moog, Neutralizing antibodies inhibit HIV-1 infection of plasmacytoid dendritic cells by an FcgammaRIIa independent mechanism and do not diminish cytokines production. *Sci Rep* **4**, 5845 (2014).

15.	C. Vandergeeten, R. Fromentin, E. Merlini, M. B. Lawani, S. DaFonseca, W. Bakeman, A. McNulty, M. Ramgopal, N. Michael, J. H. Kim, J. Ananworanich, N. Chomont, Cross-clade ultrasensitive PCR-based assays to measure HIV persistence in large-cohort studies. *Journal of virology* **88**, 12385-12396 (2014).
